# Descriptive, Exploratory Analysis of Microbial Community Composition Across Stages of Canine Periodontal Disease Revealed by 16S rRNA Sequencing in Dogs

**DOI:** 10.3390/vetsci13101016

**Published:** 2026-09-26

**Authors:** Lucas José Luduverio Pizauro, Nina Gabriela Silva Gualberto, Katharine Costa Santos, Carlos Priminho Pirovani, Juliano Oliveira Santana, Luiza Montenegro Cintra Castro, Ricardo Pedro Moreira Dias, Mónica Susana Claudino Nunes, Renata Santiago Alberto Carlos

**Affiliations:** 1 Department of Agricultural and Environmental Sciences, Santa Cruz State University, Ilhéus 45662-900, Brazil; ljlpizauro@uesc.br; 2 Graduate Program in Animal Science, Department of Agricultural and Environmental Sciences, Santa Cruz State University, Ilhéus 45662-900, Brazil; 3 Department of Biological Sciences, Santa Cruz State University, Ilhéus 45662-900, Brazil; pirovani@uesc.br; 4 Graduate Program in Genetics and Molecular Biology, Santa Cruz State University, Ilhéus 45662-900, Brazil; 5 Independent Researcher, Ilhéus 45662-900, Brazil; 6 Biosystems & Integrative Sciences Institute, Faculty of Sciences, University of Lisbon, 1749-016 Lisbon, Portugal; 7 CE3C—Centre for Ecology, Evolution and Environmental Changes, Institute for Global Change and Sustainability, 1749-016 Lisbon, Portugal

**Keywords:** dysbiosis, *Fusobacterium* sp., microbial diversity, oral microbiota, *Porphyromonas* sp., *Treponema* sp.

## Abstract

Periodontal disease (PD) is one of the most prevalent oral conditions in dogs, yet the microbial dynamics underlying its progression remain incompletely understood. This exploratory study aimed to describe the canine oral microbiome across four clinical stages using 16S rRNA gene sequencing. Dogs were categorized as Healthy (Stage 0), Gingivitis (Stage 1), Early Periodontitis (Stage 2), and Moderate–Advanced Periodontitis (Stages 3–4) according to American Veterinary Dental College (AVDC) criteria. Pooled subgingival plaque samples from the groups were analyzed for microbial diversity, taxonomic composition, and ecological patterns. Alpha diversity differed among the four pooled profiles, with the highest observed richness in the Early Periodontitis (Stage 2) pool and the lowest in the Moderate–Advanced Periodontitis (Stages 3–4) pool. Beta diversity analyses showed separation among the pooled profiles, particularly between the healthy and severe groups. Taxonomic profiling identified higher relative abundances of sequences assigned to *Porphyromonas gingivalis* and *Treponema denticola* in the more advanced disease stage pools, while *Fusobacterium nucleatum* reached its highest relative abundance in the Early Periodontitis (Stage 2) pool. Visualization of abundance profiles across the four pooled libraries revealed concordant trends among selected treponeme taxa and *Fusobacterium nucleatum*, as well as inverse trends for other taxa. These findings provide a descriptive, group-level characterization of microbial community differences across AVDC-defined stages of canine periodontal disease based on pooled samples and generate hypotheses for future individual-level studies.

## Simple Summary

Periodontal disease (PD) is one of the most common health problems affecting dogs and can lead to pain, tooth loss, and reduced quality of life if left untreated. The disease is associated with changes in the natural community of bacteria living in the mouth, but these differences are still not fully understood in dogs. In this study, we examined the oral bacterial communities of dogs with healthy mouths and compared them with dogs at different stages of PD using pooled samples. We observed differences in the composition of these bacterial communities among the clinical stages, with certain bacteria showing higher relative abundance in the more advanced disease-stage groups. These findings improve our understanding of how the canine oral microbiome differs across PD stages and provide a foundation for future studies aimed at developing better methods for early diagnosis, prevention, and treatment. A better understanding of these microbial changes may ultimately contribute to improving oral health, animal welfare, and quality of life in dogs.

## 1. Introduction

Periodontal disease (PD) is a highly prevalent, multifactorial inflammatory condition in dogs that initiates as gingivitis and progresses through varying degrees of periodontitis, leading to progressive destruction of the periodontal tissues, as a result of complex interactions between oral microorganisms and the host immune response [1,2]. Biofilm formation begins with the acquisition of a glycoprotein pellicle on the tooth surface, a normal process that occurs regardless of disease status; bacterial adherence and maturation of the resulting biofilm may remain compatible with health or, under dysbiotic conditions, progress toward disease [3]. While the clinical manifestations and risk factors of PD in dogs are well characterized, less is known about differences in microbial community composition among clinical stages of PD and the potential role of the oral microbiome in disease severity [4]. Understanding these microbial dynamics could provide key insights into disease pathogenesis and inform novel therapeutic strategies [4].

PD is now widely recognized as a polymicrobial infectious–inflammatory disease driven by dysbiotic microbial communities rather than by individual pathogens, in accordance with Socransky’s postulates and the ecological plaque hypothesis [5,6,7]. Advanced PD has been associated with microbial communities enriched in pathobionts that may contribute to inflammation and tissue destruction through complex ecological relationships described in previous studies [7]. Dental plaque formation is a dynamic and structured ecological process beginning with early colonizers adhering to the acquired pellicle, followed by the sequential recruitment of secondary colonizers and bridging species, ultimately leading to a mature and structurally complex biofilm [7]. As the biofilm matures, microbial interactions intensify, metabolic cooperation increases, and inflammatory signaling becomes amplified, creating a self-sustaining pathogenic environment [7].

The gingival sulcus and the periodontal pocket represent distinct ecological niches within the oral cavity. While the gingival sulcus in health supports a relatively stable community of commensal and symbiotic microorganisms, disease progression leads to the formation of deeper periodontal pockets characterized by reduced oxygen tension and increased nutrient availability. These microenvironmental changes strongly favor obligatory and facultative anaerobic organisms, thereby reinforcing microbial dysbiosis and promoting chronic inflammation [8].

In the healthy oral ecosystem, microbial communities exist in a state of eubiosis, characterized by functional stability and mutually beneficial host–microbe interactions [7]. PD emerges when this balance is disrupted, resulting in dysbiosis, defined by the depletion of beneficial symbionts and the expansion of pathobionts with virulence potential. This framework emphasizes that periodontal pathology is fundamentally ecological in nature, arising from community-level imbalance rather than from the mere presence of individual pathogens [7]. Dysbiosis in this context can manifest as the depletion of beneficial commensal organisms, the disproportionate expansion of pathobionts, or both, marking a shift toward compromised homeostasis and increased disease susceptibility [7].

Recent evidence also highlights the importance of the oral–gut microbiota axis, whereby oral microorganisms influence gastrointestinal microbial composition and systemic immune responses. Periodontal dysbiosis may therefore contribute not only to localized inflammation but also to systemic disease through microbial translocation, altered barrier function, and immune modulation [9,10,11]. Although this interaction remains insufficiently explored in veterinary medicine, it underscores the translational relevance of canine PD as a model for systemic inflammation and microbiota–host interactions.

Advances in next-generation sequencing have enabled more detailed characterization of the oral microbiome in both human and veterinary contexts. For example, 16S rRNA sequencing studies of aging dogs revealed that microbial profiles in older animals closely resemble those observed in dogs with PD [12]. PD in dogs has been associated with microbial dysbiosis, characterized by overgrowth of pathogenic bacteria and reduced microbial diversity [4]. In humans, key pathogens, including *Porphyromonas gingivalis*, *Fusobacterium nucleatum*, and *Treponema denticola*, are implicated in disease pathogenesis [7,8]. Despite these insights, most studies have focused on humans, and the microbial dynamics of canine PD remain poorly understood, highlighting the need for species-specific investigations.

The canine oral microbiome is highly diverse, encompassing a broad range of bacteria in both healthy and diseased states. Studies have shown clear differences between the microbiomes of healthy dogs and those with PD, with specific taxa associated with gingivitis and mild inflammation [4]. As disease progresses, the microbial community becomes dominated by anaerobic, pathogenic taxa, contributing to more severe tissue destruction [8]. Nevertheless, the precise dynamics of microbial diversity, composition, and interspecies interactions across different stages of PD remain incompletely understood. Importantly, molecular studies have demonstrated that the canine oral microbiome shares less than 20% of taxa with the human oral microbiome [13,14,15], underscoring the need to interpret canine microbiome data within a species-specific framework. Longitudinal studies have tracked community changes associated with progressive PD in dogs [16], and cross-sectional surveys have characterized subgingival microbiota across disease stages [17]. Niemiec [18] profiled oral bacterial communities across American Veterinary Dental College (AVDC)-defined disease stages using individual sample profiles, providing a directly comparable reference for the present exploratory study.

To the best of our knowledge, this is among the few studies worldwide, and one of the few from Brazil to characterize the canine subgingival microbiome across all AVDC-defined periodontal disease stages simultaneously, providing regionally grounded data within a broader framework of stage oriented microbiome research. Rather than testing predefined hypotheses, this study was designed as an exploratory, stage-oriented ecological survey to identify microbiome patterns across disease progression, generating hypotheses for future individually replicated investigations.

## 2. Materials and Methods

### 2.1. Study Location and Animal Selection

The study was conducted at the Veterinary Hospital of Santa Cruz State University (UESC), Bahia, Brazil. Subgingival plaque samples were collected from the upper teeth of dogs attending the outpatient units of the Veterinary Hospital. A total of 40 dogs were included in the study. All animals weighed up to 15 kg, regardless of sex or breed, and were between 1 and 14 years of age. The dogs were recruited during routine clinical care at the University’s Veterinary Hospital. Inclusion criteria required that the animals were fed exclusively with commercial dry food and had not received antibiotics, undergone dental treatment, or received any form of home dental hygiene in the three months prior to sampling.

### 2.2. PD Classification and Experimental Design

PD was diagnosed by a single trained member of the team (RSAC) based on oral clinical examination, periodontal probing depth, and clinical attachment level, using a calibrated millimeter periodontal probe. Following clinical evaluation, the dogs were assigned to four groups based on the classification system of the American Veterinary Dental College [19]. The dogs were assigned to one of four experimental groups based on their predominant periodontal condition:

- Healthy (Stage 0)—clinically healthy oral cavity with no gingival inflammation and no attachment loss (n = 10).
- Gingivitis (Stage 1)—gingival inflammation without attachment loss and periodontal probing depth < 3 mm, without gingival recession (n = 10).
- Early Periodontitis (Stage 2)—probing depth and attachment loss consistent with early periodontitis (<25% attachment loss), accompanied by calculus accumulation and mild gingival recession where present (n = 10).
- Moderate–Advanced Periodontitis (Stages 3–4)—attachment loss consistent with moderate (25–50%) or advanced (>50%) periodontitis, including gingival recession > 5 mm and visible root exposure where applicable (n = 10). These two stages were combined due to the limited number of dogs in each individual group, as both are considered advanced stages of PD.

Each animal was classified based on its overall clinical condition rather than requiring identical periodontal staging across all teeth. Subgingival plaque samples were collected using sterile curettes from the upper canine, premolar, and molar teeth of each animal, when present, identified according to the modified Triadan numbering system as recommended by the American Veterinary Dental College (AVDC) [canine (104, 204), premolar (105, 205, 106, 206, 107, 207, 108, 208), and molar (109, 209, 110, 210)]. Samples were immediately placed into 350 μL of TE buffer (50 mM Tris pH 7.6, 1 mM EDTA pH 8.0, and 0.5% Tween 20) and stored at −80 °C until processing.

### 2.3. Sample Pool Preparation, DNA Extraction, Library Preparation and Sequencing

Samples from all dogs within each experimental group were pooled prior to DNA extraction and sequencing, resulting in one composite microbiome profile for each disease stage. Prior to DNA extraction, samples were subjected to mechanical disruption by bead-beating to facilitate lysis of bacterial cells embedded within the plaque biofilm matrix, and then lysed in lysis buffer to release DNA, which was extracted using the DNeasy PowerSoil Kit (Qiagen, Hilden, Germany) following the manufacturer’s instructions. DNA extraction, library preparation, and purification were performed at the Center for Biotechnology and Genetics (CBG/UESC), and sequencing was conducted at the Genomics Center, University of Minnesota (USA). Extracted DNA was diluted to a concentration suitable for downstream applications.

The V3–V4 hypervariable region of the bacterial 16S rRNA gene was amplified in a 25 μL PCR reaction containing 2 μL template DNA, 12.5 μL HiFi HotStart ReadyMix (Kapa Biosystems, Wilmington, MA, USA), and 10 μL of universal primers (5 μL forward and 5 μL reverse, 10 pM each). The primer sequences were: forward 341F (5′-CCTACGGGNGGCWGCAG-3′) and reverse 805R (5′-GACTACHVGGGTATCTAATCC-3′). Thermal cycling conditions consisted of an initial denaturation at 95 °C for 3 min, followed by 25 cycles of 95 °C for 30 s, 55 °C for 30 s, and 72 °C for 30 s, with a final extension at 72 °C for 5 min. PCR products were verified by electrophoresis on a 1% agarose gel. Amplicons were purified with AMPure^®^ XP magnetic beads (Beckman Coulter, Brea, CA, USA), washed twice with 80% ethanol, and eluted in 10 mM Tris buffer (pH 8.5). Indexing was performed in a second PCR using the Nextera XT Index Kit (Illumina, San Diego, CA, USA) in a 50 μL reaction containing 5 μL purified product, 25 μL HiFi HotStart ReadyMix, and 10 μL index primers, following Illumina’s 16S Metagenomic Sequencing Library Preparation protocol. Indexed libraries were purified again with AMPure^®^ XP beads and confirmed on a 1% agarose gel. No-template negative controls were included in the amplification reactions.

Libraries were quantified by qPCR using the Kapa Library Quantification Kit (Illumina), diluted 1:1,000,000 in Tris-HCl with 0.05% Tween-20. Quantification was performed in triplicate using a six-point standard curve (20 pM to 0.0002 pM) with standards, samples, and negative controls in a 96-well plate. Final pooled libraries at 4 nM were sequenced on an Illumina MiSeq platform (2 × 300 bp paired-end) at the Genomics Center, University of Minnesota (USA), according to the facility’s standard protocols.

An overview of sample preparation, DNA extraction, library preparation, and sequencing workflow is illustrated in Figure 1.

### 2.4. Bioinformatics Analysis

Raw sequencing reads were processed using a customized BioISIGenomics^®^ analytical pipeline configured specifically for 16S rRNA amplicon sequencing data. Adapter sequences and low-quality reads were trimmed with Trimmomatic (v 0.27) [20] with the following parameters: LEADING:30, TRAILING:30, SLIDINGWINDOW:5:20.

Taxonomic classification was performed with Kraken2 (v. 2.12.0) [21] against the GenBank Bacteria and Archaea reference database (version: May 2022) [22], using a k-mer-based Lowest Common Ancestor (LCA) approach as implemented in the BioISIGenomics^®^ pipeline (BioISI, University of Lisbon, Lisbon, Portugal). Default classification parameters of the pipeline were applied.

Rarefaction curves were generated to assess sequencing depth sufficiency; no rarefaction to equal depth was applied prior to downstream analyses. All diversity metrics were computed using relative abundance data and should be interpreted as descriptive rather than inferential.

Operational Taxonomic Units (OTUs) were clustered at 97% sequence similarity. Only taxa with a minimum relative abundance threshold of 0.01 at the genus level were retained for downstream analyses. OTU clustering at 97% similarity was selected to maintain compatibility with the BioISIGenomics^®^ k-mer-based LCA classification framework, which operates at read-level taxonomic assignment rather than denoised sequence variants. The range of 356–392 OTUs reported for each pooled library corresponds to the number of OTUs returned by the BioISIGenomics^®^ analytical pipeline after application of the 0.01 relative-abundance filter at the genus level. In contrast, the higher observed-richness values (420–563 OTUs) were calculated directly from the full OTU table and include all taxa detected with at least one read, prior to filtering.

Group-level variation was visualized with heatmaps based on Bray–Curtis dissimilarity and hierarchical clustering using Ward’s linkage method, as implemented in the scipy.cluster.hierarchy module (SciPy1.18.1). Alpha diversity was estimated using the Shannon diversity index, and beta diversity was assessed by Principal Coordinates Analysis (PCoA) based on Bray–Curtis distances [23]. Phylogenetic relationships and taxonomic flows across disease stages were illustrated using Sankey flow diagrams generated from raw OTU counts [24].

### 2.5. Taxonomic Classification

Taxonomic names are reported using the original nomenclature from the reference database to maintain consistency with figures, tables, and analytical outputs. When applicable, updated taxonomic names based on recent prokaryotic taxonomic revisions are provided at first mention in parentheses for clarity, without altering the original labeling (e.g., Proteobacteria = *Pseudomonadota*, Firmicutes = *Bacillota*, Bacteroidetes = *Bacteroidota*, Spirochaetes = *Spirochaetota*, Fusobacteria = *Fusobacteriota*). This classification is intended solely for alignment with current nomenclature used in contemporary literature and was not applied as a reclassification step [25].

### 2.6. AI Usage Disclaimer and Figure Preparation

All figures were prepared and manually edited in Inkscape 1.4.4. ChatGPT (GPT—4, OpenAI) was used as an AI-assisted tool for copyediting text to improve readability, grammar, and scientific style. Methodology pictograms were generated and assembled in Inkscape. Microbiome analysis visualizations were produced in R and subsequently compiled in Inkscape. AI tools were not used for data analysis, taxonomic classification, sequence processing or the generation of scientific conclusion; all analytical and interpretative work was performed by the authors.

## 3. Results

### 3.1. Study Cohort

A total of 40 dogs (22 females and 18 males) were enrolled in this study, with body weights ranging from 2.60 to 15.0 kg (median: 7.50 kg). The cohort included multiple breeds, comprising mixed-breed dogs (n = 16), Pinscher (n = 8), Poodle (n = 6), Shih Tzu (n = 4), Shar-Pei (n = 2), Labrador Retriever (n = 2), Cocker Spaniel (n = 1) and Yorkshire Terrier (n = 1) (Table 1).

Notably, this study included all AVDC-defined stages of PD simultaneously. Due to clinical recruitment constraints, age matching between groups was not implemented. Consequently, the Healthy and Gingivitis groups consisted predominantly of younger dogs (1–4 years), whereas the Early and Moderate–Advanced Periodontitis groups included older animals (6–14 years) (Table 1).

### 3.2. Sequencing, Rarefaction Curves, Alpha Diversity, and Beta Diversity

The number of raw sequencing reads per pooled library ranged from 67,552 to 181,313. In descending order, the Early Periodontitis (Stage 2) pool yielded the highest number of reads (181,313), followed by Moderate–Advanced Periodontitis (Stages 3–4) (128,115), Gingivitis (Stage 1) (79,287), and Healthy (Stage 0) (67,552).

Across groups, between 356 and 392 OTUs were identified per pooled library. Observed richness (including low-abundance OTUs detected across pooled profiles) ranged from 356 to 563 OTUs across groups (per-group values reported below). Rarefaction curves (Figure 2A) reached a plateau for all pooled libraries, indicating that sequencing depth was sufficient to capture microbial richness at the pooled level.

Alpha diversity metrics for pooled profiles are shown in Figure 2B,C. Observed richness was 549 OTUs for Healthy (Stage 0), 461 OTUs for Gingivitis (Stage 1), 563 OTUs for Early Periodontitis (Stage 2), and 420 OTUs for Moderate–Advanced Periodontitis (Stages 3–4). Shannon diversity indices were 4.73, 4.15, 4.25, and 4.30 for Stages 0, 1, 2, and 3–4, respectively. Because libraries were generated from pooled DNA extracts rather than individual samples, diversity metrics are presented as descriptive summaries rather than subjected to statistical comparison.

Beta diversity was explored using Principal Coordinates Analysis (PCoA) based on Bray–Curtis dissimilarity (Figure 2D). The first coordinate (PCoA1) explained 64.5% of the variance and revealed a gradient across AVDC disease stages. Pooled profiles from Healthy (Stage 0) clustered to the left, followed sequentially by Gingivitis (Stage 1) and Early Periodontitis (Stage 2), while samples from Moderate–Advanced Periodontitis (Stages 3–4) clustered to the right. This distribution indicates differences among the four pooled profiles, with separation particularly between the Healthy and Moderate–Advanced Periodontitis groups. Differences are presented descriptively, as statistical inference is not appropriate for pooled samples.

### 3.3. Differences in Microbial Community Composition Across PD Severities

The Healthy (Stage 0) pooled profile exhibited a balanced microbial community dominated by Proteobacteria (Pseudomonadota; 29.8%), Bacteroidota (27.5%), and Bacillota (20.0%). Bacteroidota was higher in the Gingivitis (Stage 1), Early Periodontitis (Stage 2), and Moderate–Advanced Periodontitis (Stages 3–4) pools (37.9%, 36.0%, and 38.1%, respectively) than in the Healthy pool. Spirochaetota was also higher in the more advanced disease-stage pools, increasing from 4.5% (Stage 0) to 14.1% (Stages 3–4), along with higher relative abundance of Fusobacteriota (7.4% to 8.3–10.1%).

Higher relative abundances of Bacteroidia (33.7–36.6%), Spirochaetia (9.7–14.1%), and Fusobacteriia (8.3–10.1%) were observed in the disease-stage pools, while Clostridia and Gammaproteobacteria remained relatively stable or were lower.

Bacteroidales, Spirochaetales, and Fusobacteriales showed higher relative abundances in the more advanced disease-stage pools. Bacteroidales increased from 21.1% (Stage 0) to 35.7% (Stages 3–4), and Spirochaetales from 4.5% to 14.1%. In contrast, Clostridiales remained comparatively stable.

Porphyromonadaceae and Spirochaetaceae increased across disease stages. At the genus level, Porphyromonas and Treponema showed higher relative abundances in the more advanced disease-stage pools (Porphyromonas from 12.3% in Stage 0 to 18.6% in Stages 3–4; Treponema from 4.2% to 13.4%). Fusobacterium reached its highest relative abundance (7.51%) in the Early Periodontitis (Stage 2) pool.

Species-level assignments from 16S rRNA sequencing represent putative identifications based on sequence similarity and should be interpreted with caution. Sequences assigned to *Porphyromonas gingivalis* (putative identification based on 16S V3–V4 similarity) were higher in the Moderate–Advanced Periodontitis pool (7.99%) than in the Healthy pool (3.61%). *Fusobacterium nucleatum* reached its highest relative abundance in the Early Periodontitis pool (7.49%), and *Treponema denticola* reached 6.58% in the Stages 3–4 pool (Figure 3).

To investigate compositional changes across PD stages, a Z-score heatmap based on relative taxon abundance was generated (Figure 4). Columns represent AVDC-defined clinical stages—Healthy (Stage 0), Gingivitis (Stage 1), Early Periodontitis (Stage 2), and Moderate–Advanced Periodontitis (Stages 3–4)—and rows represent individual taxa clustered by similarity in abundance pooled profiles. Color intensity reflects Z-scores, where darker shades suggests higher relative abundance relative to each taxon’s mean across groups.

Differences in microbial composition were observed among pooled profiles of AVDC clinical stages. Notably, *Fusobacterium nucleatum*, *Porphyromonas gingivalis*, and *Treponema denticola* showed higher relative abundance in the moderate and advanced disease-stage pools compared with the healthy and gingivitis pools. Conversely, taxa such as *Moraxella lacunata*. were more abundant in Early Periodontitis (Stage 2) and declined thereafter.

Several taxa, including *Acholeplasma axanthum* and *Bacteroides uniformis*, occurred across all stages with limited variation, suggesting broader ecological distribution. In contrast, *Campylobacter rectus*, and *Clostridioides difficile* showed greater representation in Gingivitis (Stage 1) and Early Periodontitis (Stage 2), with lower abundance in later stages, a pattern consistent with descriptive composition shifts across the pooled disease-stage profiles.

### 3.4. Similarity of Taxon Abundance Profiles

To explore distribution patterns across stages, abundance trends were visualized using relative abundance from the four pooled libraires (Figure 5). Because samples were pooled and lacked biological replication, these patterns were not interpreted statistically. Color gradients represent concordant or opposing abundance trends across AVDC stages. Clustering among *Treponema vincentii*, *T. maltophilum*, *T. denticola*, and *F. nucleatum* reflects similar abundance trends across the four pooled libraries, while inverse trends among other taxa indicate contrasting distribution patterns. All results are descriptive and exploratory.

## 4. Discussion

This study provides a descriptive ecological characterization of differences in microbial community composition across AVDC-defined stages of PD pooled profiles in dogs, including diversity metrics, taxonomic composition, and concordant abundance trends. To the best of our knowledge, this is one of the few Brazilian studies to characterize the canine oral microbiome across all four AVDC-defined stages simultaneously. Including ten dogs per group ensured an equal distribution of samples across disease categories. The 16S rRNA sequencing approach enabled broad taxonomic profiling across multiple hierarchical levels.

Distinct microbial profiles were observed among the four pooled disease-stage libraries. Because samples were pooled by stage prior to DNA extraction and sequencing, only group-level community profiles could be generated. Consequently, inter-individual variation could not be assessed, and all interpretations are exploratory and descriptive rather than inferential. Accordingly, taxa described as enriched in specific disease stages should be interpreted as characteristics of pooled community profiles rather than biomarkers consistently associated with individual animals. Also, the OTU clustering at 97% similarity was selected to maintain compatibility with the BioISIGenomics^®^ k-mer-based LCA classification framework, which operates at read-level taxonomic assignment rather than denoised sequence variants. While ASV-based approaches offer improved resolution and cross-study reproducibility, OTU-based clustering remains appropriate for exploratory, pooled-sample designs where individual-level variance cannot be assessed.

### 4.1. Microbial Diversity Patterns and Community Composition Across PD Stages

Alpha diversity varied among the four pooled profiles. Observed richness was highest in the Early Periodontitis (Stage 2) pool and lower in the Moderate–Advanced Periodontitis (Stages 3–4) pool. These differences are reported descriptively only.

Differences in diversity among the pooled profiles may reflect changes in ecological conditions associated with different clinical stages. Similar patterns of diversity variation have been reported in human periodontal disease studies.

Shannon diversity decreased from healthy to diseased stages, with partial recovery in advanced disease. This suggests early dominance by a subset of pathogens followed by a compositional profile distinct from that of healthy dogs. Similar patterns have been reported in human PD, where functional redundancy and resilience emerge within a disease-adapted microbiome [7].

### 4.2. Taxonomic Composition and Putative Periodontal Pathogens

Taxonomic profiling of the pooled libraries showed higher relative abundances of Spirochaetes, Fusobacteria, and Bacteroidetes in the more advanced disease-stage pools, consistent with previous reports describing anaerobe-enriched microbial communities associated with periodontal inflammation. At the genus level, Porphyromonas, Treponema, and Fusobacterium expanded markedly as disease advanced.

Sequences assigned to *Porphyromonas gingivalis* were higher in the advanced disease-stage pool (7.99%) than in the Healthy pool (3.61%). This species has been described as a keystone pathogen in the periodontal literature [6,7]. Inflammatory conditions favor pathogens with enhanced proteolytic capacity, immune evasiveness, and quorum sensing capability, allowing them to manipulate host signaling networks and destabilize microbial homeostasis [26].

Higher relative abundances of sequences assigned to *Treponema denticola* and *Fusobacterium nucleatum* were observed in specific disease-stage pools, consistent with previous reports describing these taxa in canine and human periodontal disease [27,28].

### 4.3. Study Limitations

Pooling limited biological resolution. This approach collapsed heterogeneous individual microbiomes into a single composite profile per disease stage, masking inter-individual variability, reducing the detection of rare taxa, and increasing the apparent dominance of highly abundant organisms. Consequently, the findings should be interpreted as descriptive community-level patterns rather than evidence of taxa consistently associated with periodontal disease at the individual level. Pooling was adopted because financial constraints precluded sequencing each sample individually, allowing an initial exploratory characterization of microbial community composition across disease stages.

In addition, the modest sample size (40 dogs) and single-center recruitment at one veterinary hospital limit the generalization of these findings. Assembling a larger, multi-center cohort was not feasible within the resources available for this study, given the costs of radiographic staging and microbiological/sequencing analyses in Brazil. Comparably sized cohorts have nonetheless been used in other oral microbiome studies, including investigations of periodontal pathogens in dogs [29], and the present work should be interpreted as an exploratory, regionally grounded characterization rather than a broadly generalizable survey.

Age differences among disease-stage groups should also be interpreted in light of the natural history of periodontal disease rather than as an independent confound introduced by the sampling design. Periodontal disease severity is well known to increase with age in dogs that do not receive regular dental care; accordingly, dogs were recruited on the basis of their AVDC-defined stage, not age, and the predominance of younger animals in the Healthy and Gingivitis groups and of older animals in the Early and Moderate–Advanced Periodontitis groups reflects this established age–severity association. Eliminating this association without artificially restricting the disease spectrum in each group was not feasible. Consequently, the present cross-sectional design cannot fully disentangle disease associated microbial changes from age associated changes, and this should be considered when interpreting the reported association between periodontal stage and microbiome composition.

Recent transcriptomic studies in feline oral disease have demonstrated that inflammatory signatures and host–microbe interactions are only resolved when individual variation is preserved [30]. Future canine studies should adopt individual-level sampling and ideally integrate multi-omics approaches to dissect host–microbe crosstalk.

The GenBank Bacteria and Archaea reference database (2022), used here via Kraken2 within the BioISIGenomics^®^ pipeline, is not specifically curated for 16S rRNA amplicon sequencing. Databases such as SILVA or Greengenes2 contain more comprehensive coverage of canine oral taxa and are more widely used in amplicon-based microbiome research. This may explain the identification of unusual environmental taxa (e.g., *Nitratiruptor* sp., *Geofilum rubicundum*) which originate from deep-sea hydrothermal vents unlikely to inhabit the canine oral cavity, whose detection most likely reflects misclassification due to limited canine oral representation in the reference database. Future studies should employ 16S-specific curated databases and, ideally, canine-adapted oral microbiome references.

### 4.4. Study Scope and Interpretation

Because biological replication was not available, no conclusions can be drawn regarding causality, diagnostic performance, or predictive utility. Results reflect group-level ecological patterns only and should not be extrapolated to individual animals. This study is therefore best interpreted as an exploratory ecological survey rather than a diagnostic or intervention-focused investigation.

## 5. Conclusions

This exploratory study provides a descriptive characterization of the canine oral microbiome across all four AVDC defined-stages of periodontal disease based on pooled subgingival plaque samples. As one of the few Brazilian studies to examine the full spectrum of AVDC stages simultaneously, it contributes regionally grounded data to the veterinary microbiome literature. Alpha diversity varied among the four pooled profiles; these differences are reported descriptively only, given the absence of biological replication. Distinct microbial profiles were observed across the pooled disease-stages libraries, including higher relative abundance of sequences assigned to *Porphyromonas gingivalis* and *Treponema denticola*, in the more advanced stages, and a peak in relative abundance of *Fusobacterium nucleatum* in the Early Periodontitis (Stage 2) pool. Abundance-profile visualizations further illustrated differences in community composition among the four libraries, including concordant trends among selected taxa and inverse trends for others. Beyond the classic periodontal pathogens, the Healthy (Stage 0) pool was characterized by a balanced community dominated by Pseudomonadota, Bacteroidota, and Bacillota, whereas the disease-stage pools showed expansion of Bacteroidales, Spirochaetales, and Fusobacteriales, together with taxon-specific shifts. Together, these findings describe group-level differences in microbial community composition across clinical stages of canine PD and provide a foundation for future hypothesis-driven studies using individual-level sampling.

## Figures and Tables

**Figure 1:**
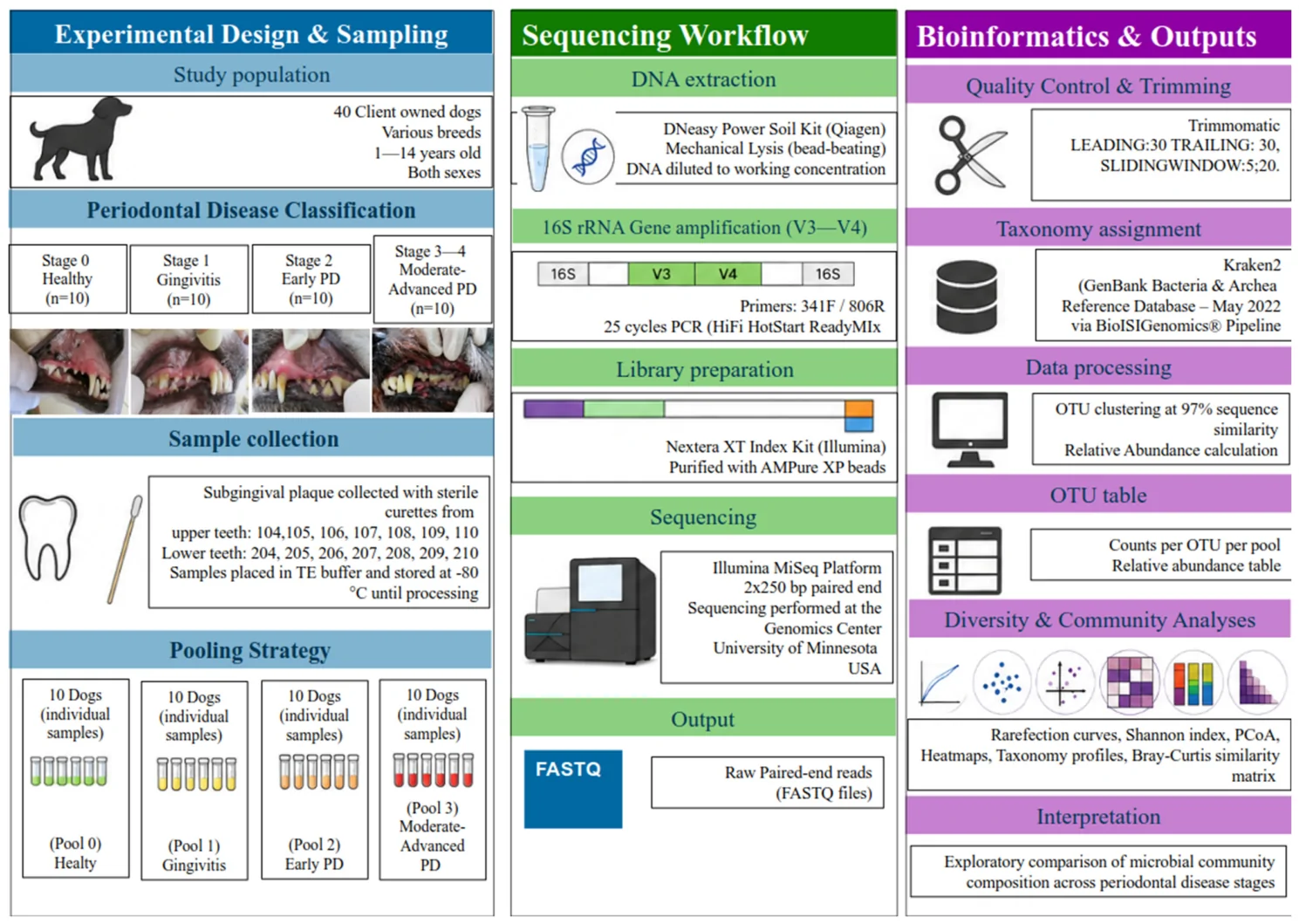
Overview of Methodology Workflow.

**Figure 2:**
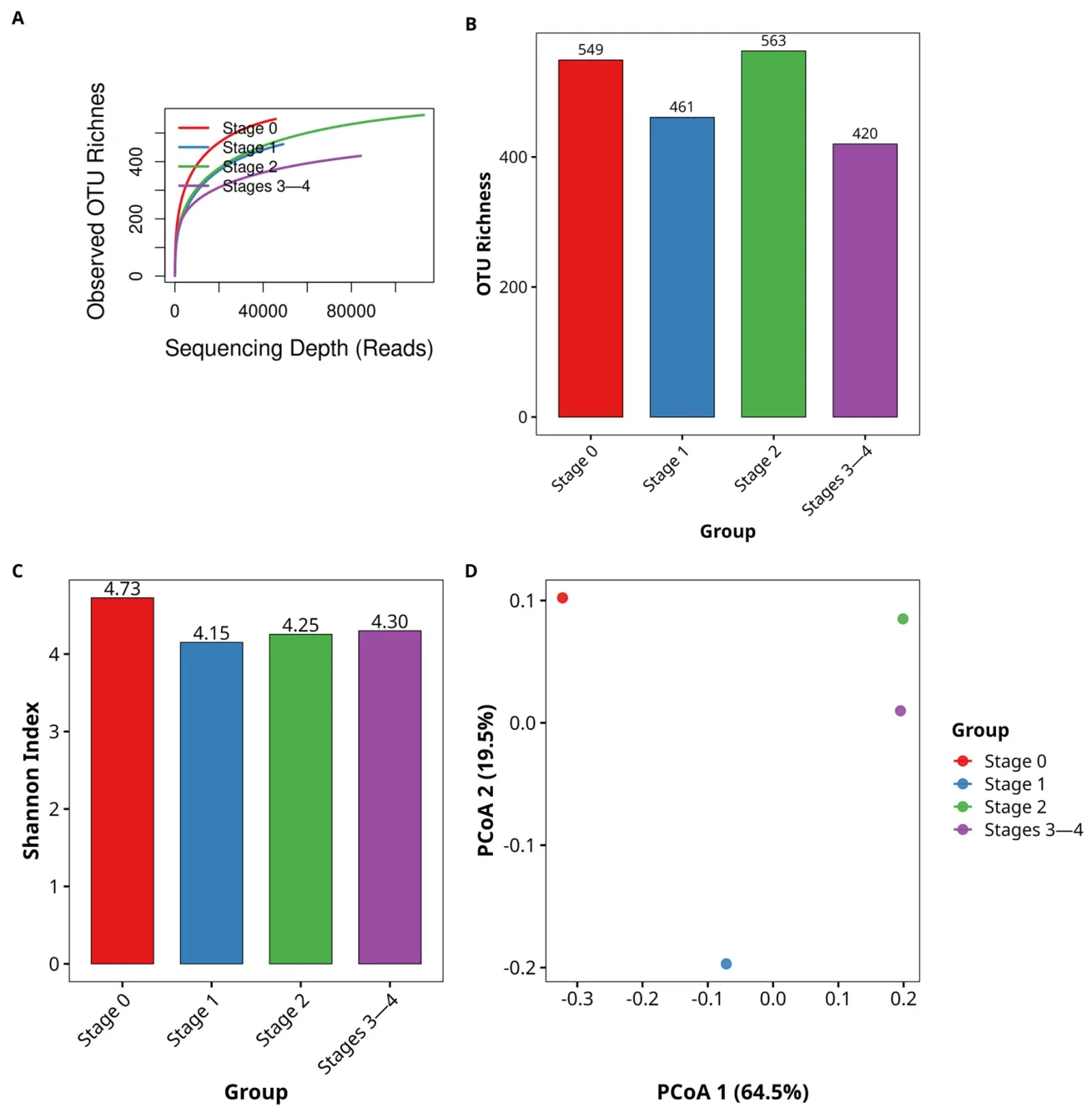
Rarefaction Curves, Alpha Diversity, and Beta Diversity of the Canine Oral Microbiome. (**A**) Rarefaction curves for each pooled library, showing sequencing depth sufficiency across AVDC stages. (**B**) Observed OTU richness for each pooled profile. (**C**) Shannon diversity index for each pooled profile. (**D**) Principal Coordinates Analysis (PCoA) based on Bray–Curtis dissimilarity, illustrating beta diversity among pooled profiles.

**Figure 3:**
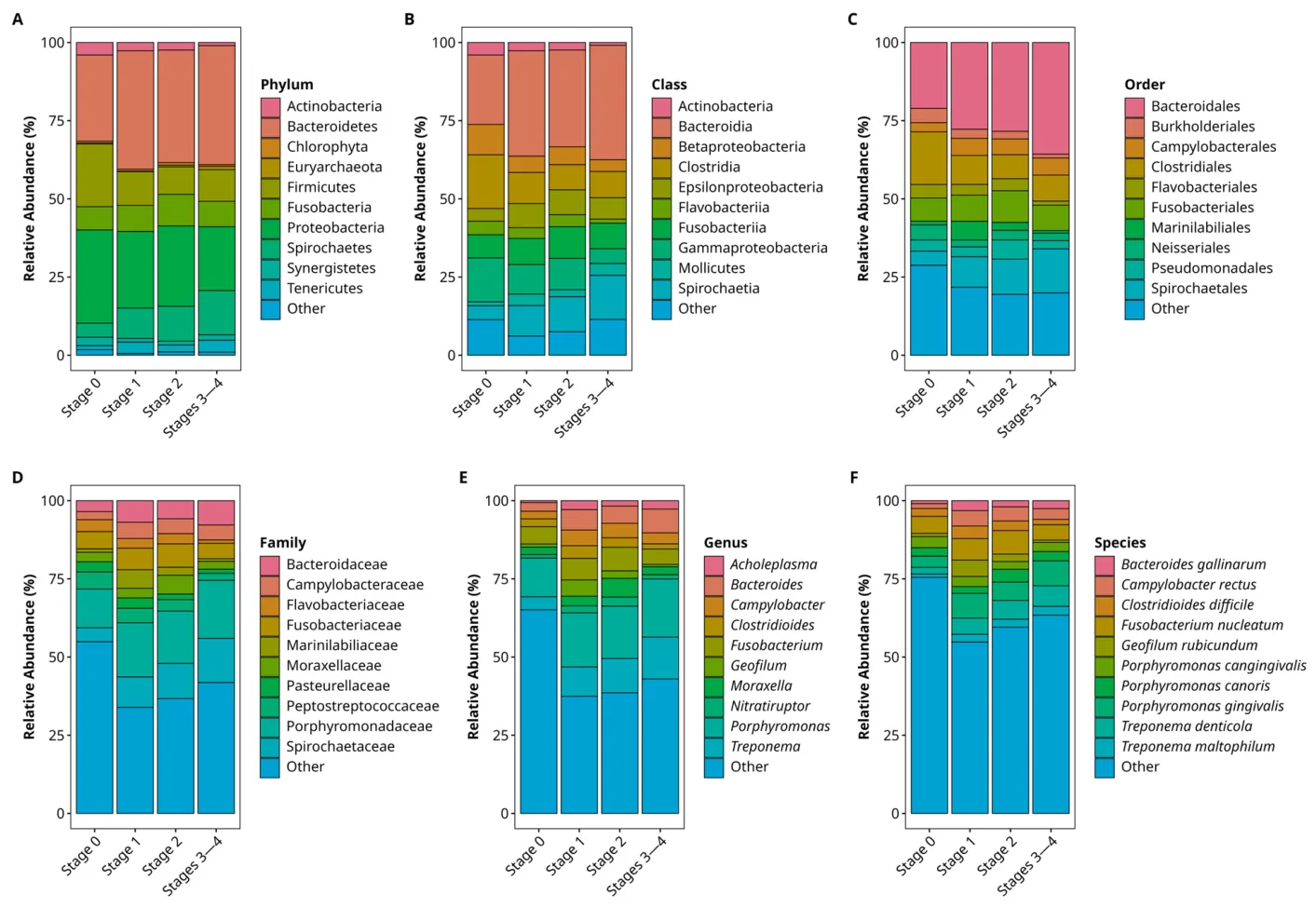
Taxonomic Composition of Microbial Communities Across PD Stages.

**Figure 4:**
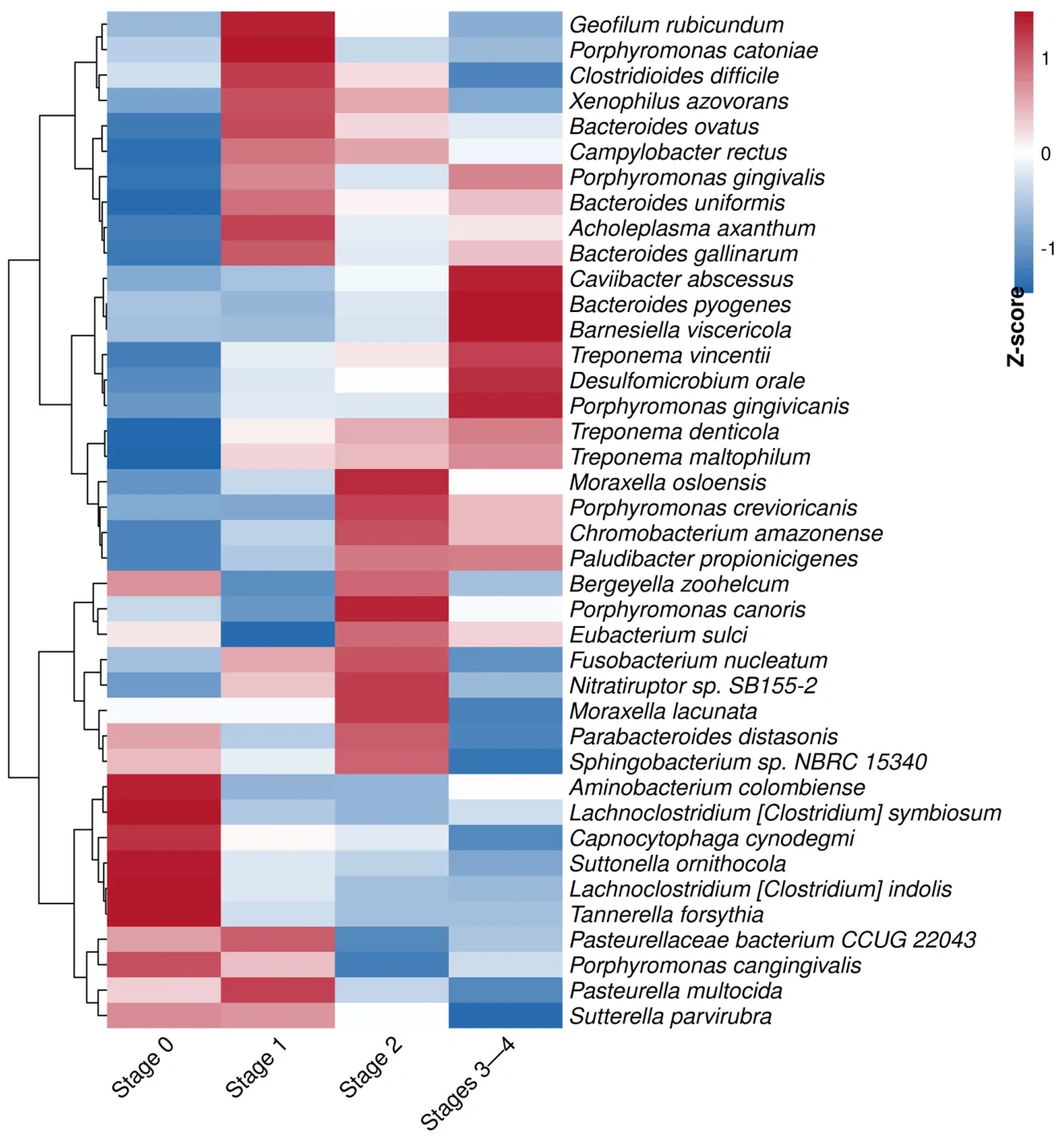
Heatmap of Bacterial Taxa Relative Abundance (Z-scores) Across Periodontal Disease Severities Based on Hierarchical Clustering Using Ward’s Linkage Method.

**Figure 5:**
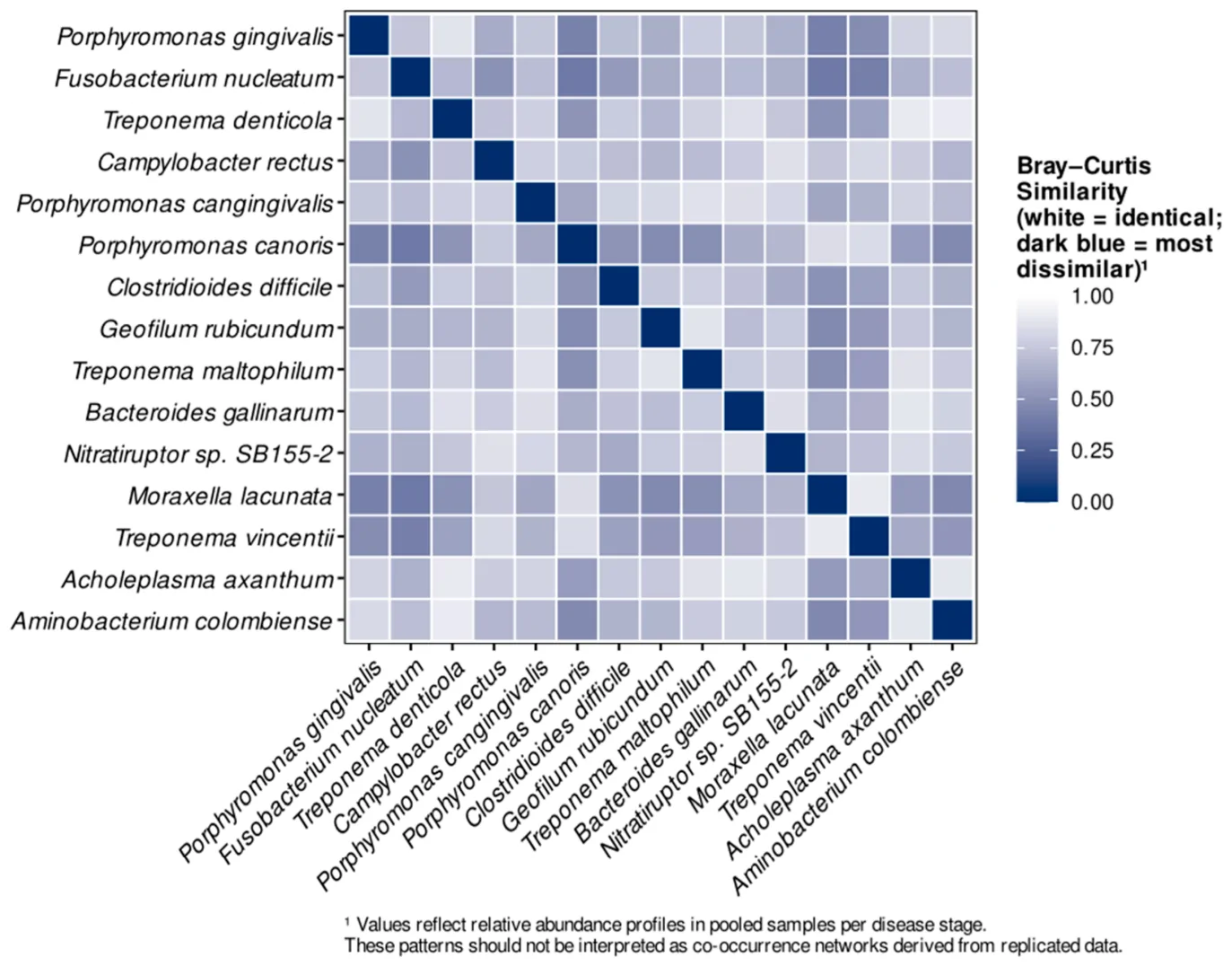
Similarity of taxon abundance profiles across the four pooled disease-stage libraries—Healthy, Gingivitis, Early Periodontitis, and Moderate–Advanced Periodontitis—based on relative abundance data from pooled subgingival plaque samples. Color gradients indicate concordant or opposing abundance trends among taxa across the four stages. Because samples were pooled and no biological replicates were available, these patterns reflect descriptive co-distribution of taxon abundance rather than statistically confirmed microbial co-occurrence or interaction.

**Table 1:** Demographic characteristics of 40 dogs included in the study according to periodontal disease stage.

Characteristic	Healthy (Stage 0)	Gingivitis (Stage 1)	Early PD (Stage 2)	Moderate–Advanced PD (Stages 3–4)
Dogs per group, n	10	10	10	10
Age, years, median (range)	1.5 (1.0–2.0)	2.3 (1.4–3.7)	6.0 (4.0–10.0)	12.5 (6.0–14.0)
Body weight, kg, median (range)	7.4 (2.6–15.0)	7.5 (2.9–12.4)	7.4 (3.0–15.0)	7.5 (2.8–13.4)
Male sex, n (%)	5 (50)	5 (50)	3 (30)	5 (50)
Female sex, n (%)	5 (50)	5 (50)	7 (70)	5 (50)
Mixed-breed dogs (SRD), n (%)	5 (50)	6 (60)	2 (20)	3 (30)
Purebred dogs, n (%)	5 (50)	4 (40)	8 (80)	7 (70)

## Data Availability

The raw sequencing reads have been deposited in the NCBI Sequence Read Archive (SRA) under BioProject ID PRJNA1478504 and are publicly available. Any additional data supporting the findings of this study, beyond what is deposited in the SRA, are available from the corresponding author upon request, in accordance with the journal’s data availability policy.
